# Advanced CKD detection through optimized metaheuristic modeling in healthcare informatics

**DOI:** 10.1038/s41598-024-63292-5

**Published:** 2024-06-01

**Authors:** Anas Bilal, Abdulkareem Alzahrani, Abdullah Almuhaimeed, Ali Haider Khan, Zohaib Ahmad, Haixia Long

**Affiliations:** 1 College of Information Science and Technology, Hainan Normal University, Haikou, 571158 China; 2 Key Laboratory of Data Science and Smart Education, Ministry of Education, Hainan Normal University, Haikou, 571158 China; 3 Computer Science Department, Faculty of Computing and Information, Al-Baha University, 65779 Al-Baha, Saudi Arabia; 4 Digital Health Institute, King Abdulaziz City for Science and Technology, 11442 Riyadh, Saudi Arabia; 5 Department of Software Engineering, Faculty of Computer Science, Lahore Garrison University, Lahore, 54000 Pakistan; 6 Department of Criminology and Forensic Sciences, Lahore Garrison University, Lahore, 54000 Pakistan

**Keywords:** Binary Grey Wolf optimization algorithm (BGWO), Extreme learning machine (ELM), Chronic kidney disease (CKD) diagnosis, Feature optimization, Classifier, Biomarkers, Health care, Medical research, Signs and symptoms

## Abstract

Data categorization is a top concern in medical data to predict and detect illnesses; thus, it is applied in modern healthcare informatics. In modern informatics, machine learning and deep learning models have enjoyed great attention for categorizing medical data and improving illness detection. However, the existing techniques, such as features with high dimensionality, computational complexity, and long-term execution duration, raise fundamental problems. This study presents a novel classification model employing metaheuristic methods to maximize efficient positives on Chronic Kidney Disease diagnosis. The medical data is initially massively pre-processed, where the data is purified with various mechanisms, including missing values resolution, data transformation, and the employment of normalization procedures. The focus of such processes is to leverage the handling of the missing values and prepare the data for deep analysis. We adopt the Binary Grey Wolf Optimization method, a reliable subset selection feature using metaheuristics. This operation is aimed at improving illness prediction accuracy. In the classification step, the model adopts the Extreme Learning Machine with hidden nodes through data optimization to predict the presence of CKD. The complete classifier evaluation employs established measures, including recall, specificity, kappa, F-score, and accuracy, in addition to the feature selection. Data related to the study show that the proposed approach records high levels of accuracy, which is better than the existing models.

## Introduction

CKD is a severe health concern in the world, affecting a combination of disorders that heavily impact renal structure and function. Despite an increased risk of issues across other organ systems, modest disparities in urine metrics and quality have been linked to an elevated chance of the disease^1,2^. Renal disease is methodically divided into four separate phases:

- CKD (Chronic Kidney Disease)
- NKD (NO Kidney Disease)
- AKI Kidney Injury is a type of (acute kidney injury)
- ESKD (End-stage kidney ailment)

The research aims to diagnose chronic kidney disease and identify individuals with N o kidney disease. CKD affects about 10% of the population, killing millions every year. Hypertension, obesity, family background, medicines, age, and race all have something to do with CKD. The medical sector has an urgent and escalating need for a method to detect CKD as soon as possible^3^.

Chronic low back issues have a variety of covered conjunctions. The escalating frequency of CKD and the complex nature of its causal agents underscore the need for better diagnostic methods. Even though we have learned much about the disease, a notable difference between early detection and precise diagnosis still exists. Even standard diagnostic techniques can occasionally be based too late in the brain to see a particular illness. In the medical sector, late notice of disease equals late treatment, and poor treatment leads to the patient more often than not. Furthermore, many factors contribute to the complexity of CKD, including imbalances in hormonal and environmental systems that can affect the disease in myriad ways. Artificial intelligence can be extremely beneficial because it has been developed for different Machine learning and can be effective in increasing the detection and diagnostic performance of CKD prediction and diagnostic performance^4–6^.

Data mining optimization has helped a lot in developing MDC and improving the healthcare sectors, largely in accurately detecting CKD^7,8^. ML technologies have been designed to boost the Medical Imaging, screening, diagnosis, and tracking of new activities. The typical disadvantage of these models is the complexity of their nature. Many of these models can not account for their estimations^9,10^. Researchers are thus turning their attention to the development of ML models capable of making explicit deliberations and supporting their judgment^11–15^. For a long time, neural networks have been acknowledged as excellent devices supporting medical decision-making. One of their advantages is that they "could improve the efficiency in managing labor-intensive activities." Besides, these systems can successfully manage datasets that "could be incomplete or noisy." Certainly, classification is one of the widespread methods in machine learning that significantly impacts "classifying data items" and ensures proper organization of health records^16–19^. This research has taken an all-embracing and holistic approach in response to contemporary research's key obstacles and limitations. More specifically, the primary focus of our endeavors is to create a diagnostic tool for CKD that effectively employs AI and ML capabilities. One of the well-documented challenges of machine learning is that it is not transparent. In other words, multiple systems may operate as "black boxes" and accurately and efficiently generate predictions but neglect to provide insight into the process. This constraint is bypassed by designing ML algorithms that have this dual feature of providing accurate judgment while simultaneously being transparent in their decision-making. A vast number of dimensions usually characterize CKD datasets. Accordingly, when analyzing these datasets, it is imperative to prioritize utilizing effective feature selection algorithms. The scientific literature witnesses the research community's propensity toward applying two main Swarm Intelligence methods, Ant Colony and Particle Swarm Optimization, to find the ultimate feature subset selection^20–24^.

This article presents the first-ever classification strategy solely created to diagnose CKD. The methodology used in this study's scope starts with the stringent data preparation step to correct missing values. In the subsequent steps, we utilize Binary Grey Wolf Optimization, a metaheuristic technique inspired by the fascinating behavior of grey wolves^25^. As we will also demonstrate, the engagement of this algorithm plays a crucial role in determining the most relevant set of characteristics, therefore boosting the accuracy of the diagnosis. Moreover, our research substantially endorses the utilization of ELM as a powerful technique for identifying CKD. The precision of our model is attested by the results of thorough testing on a standard CKD dataset, proving its robustness and predictability.

The paper has been precisely arranged to assist the readers' understanding. The report opens by giving an overview of CKD detection and detailing the research goals of our investigation. Section “Literature review” comprehensively analyzes the existing approaches used to diagnose CKD. The third portion of this study examines the suggested method for chronic CKD identification, followed by a comprehensive review of the algorithm's performance in the fourth section. Section “Discussion” of the study concludes with a thorough summary, including our primary contributions and conclusions, while suggesting prospective avenues for further research.

## Literature review

The accurate prognosis of CKD remains a significant difficulty within the healthcare field. Throughout the years, numerous researchers have devoted their endeavors to investigating various methodologies. The group's primary objective is to enhance the precision and timeliness of CKD detection. This is primarily achieved through refining data pre-processing techniques, feature selection methods, and classification approaches. AlMuhaideb and Menai^26^ adopted a different method, emphasizing the importance of pre-processing techniques. The research conducted by the authors insisted on the significance of feature subset selection and the complexities associated with managing missing data. By utilizing an ant colony metaheuristic approach in classification, the researchers could attain improved levels of predicted accuracy, occasionally exceeding 60%. However, the issue of ensuring model transparency and interpretability persisted.

Akben^27^ achieved notable progress by integrating methodologies for medical data classification, utilizing K-Means for the pre-processing process, and implementing classification techniques such as KNN, SVM, and NB. The method used by the researchers relied on assessing urine test features, resulting in a significant accuracy rate of 97.8%, especially among individuals aged 35 years and older. The current investigation has indicated that various combinations of dataset characteristics provided variable degrees of accuracy, ranging from 83.75 to 97.8%. Additionally, it should be emphasized that certain combinations took additional processing time. Yang et al.^28^ studied the implementation of the Iterative Dichotomiser 3 (ID3) approach, which applies a heuristic way to simplify the data categorization process. Although the ID3 technique showed promise, it had significant drawbacks, notably the necessity to divide dataset values. In their study^29^, Shen et al. proposed the SVssM-FOA method, which performs better than artificial neural networks (ANNs) across multiple metrics. Nonetheless, the underlying issue of maintaining model transparency and addressing datasets with numerous dimensions persisted.

Khamparia et al.^20^ introduced a unique approach that employs a deep stacked autoencoder (DSAE) technology to identify the categorization of CKD by applying multimedia data learning methodologies. The study aimed to foresee CKD during its earliest phases, helping lower treatment expenditures for afflicted persons. The current work introduces the Deep Stacked Autoencoder (SAE) method as a novel strategy for recognizing CKD. The Stacked Autoencoder (SAE) design has two auto encoders and a softmax classifier. Nevertheless, a single-stacked auto encoder proved ineffective in lowering the input features' dimensionality.

Moreover^30^,developed a prediction model for CKD using numerous resampling methods and ML systems. The resampling methods include the synthetic minority oversampling technique and Borderline-SMOTE, while the classifiers include the decision tree, AdaBoost, and random forest. The finding adopted indicates that the decision tree combining the SMOTE method exhibited the highest level of presentation, with an accuracy rate of 98.99%. Ordinarily, ML research studies adopt various attributes, such as hemoglobin level, albumin, red blood cell count, white blood cell count, blood pressure, packed cell volume, specific gravity, and others, in identifying patients at high risk of CKD. In turn, the patients can receive timely and cost-effective medical intervention from the medical practitioners. Although much focus has been given to machine learning in predicting CKD, few studies explicitly acknowledge identifying the key traits required to realize CKD detection^31–33^. If accurately identified in individuals suspected of having CKD, these characteristics could be employed for efficient computer-assisted CKD diagnosis.

The findings of Salekin and Stankovic^34^ were supported by their investigation into feature selection, where they utilized wrapper and filter procedures. In the research^35^, Elhoseny et al. directed a comprehensive examination of an investigative classification and estimate framework for chronic renal ailment. The introduction of the Density-based feature selection with the Ant Colony-based Optimization (D-ACO) method aimed to address the identification and classification of CKD inside healthcare services. The acquired performance measurements estimated the parameters and improved the overall efficacy. The primary drawbacks of this optimization approach were the occurrence of early convergence and a lack of improvement in output. In this study^36^, the authors enhance CKD diagnosis through classification and efficient feature selection methodologies. The methodology described in the study involves the application of the Oppositional FireFly Optimization (OFFO) algorithm to discover relevant features. These features are combined with a Deep Neural Network (DNN) for classification. The article highlights the significant presentation of the deep neural network (DNN) model, which achieved an impressive accuracy of 98.89% in classifying CKD.

In the study^37^, the authors employed a thorough methodology by utilizing various ML procedures in a dataset of 400 patients. The authors' methodology, which encompassed replacing missing values and fine-tuning parameters, exhibited promising results. Nevertheless, the most extensive obstacles to the transparency of models and the management of complex datasets have yet to be resolved. In a related study, the authors^38^ examined the application of Bayesian networks in identifying at-risk pregnancies. In contrast, another study by the authors^39^ focused on predicting illness comorbidities through weighted association rule mining. Although not directly associated with CKD, both research emphasized the adaptability of machine learning.

In their study, Jongbo et al.^40^ analyzed the utilization of ensemble techniques, specifically bagging and random subspace, in constructing a diagnostic model for CKD. The primary goal of the case study was to increase the classification accuracy of the Decision Tree, Naïve Bayes, and k Nearest Neighbors classifiers. A notable result was achieved with the K-nearest neighbors classifier making precise predictions using a random subspace ensemble. Meanwhile, the increased cost may be related to the necessity of comprehensive storage of healthcare training samples. The authors cited in references^41–43^ have made notable contributions by introducing distinct approaches and algorithms to improve the prediction of CKD. Nevertheless, the fundamental obstacles to high-dimensional datasets' computational complexity, interpretability, and management persisted.

Within the domain of medical data classification, where phases such as feature selection and classification have significant importance, this study explores the complexities associated with classification algorithms and feature selection techniques. The issues encountered in the prediction of CKD are complex and diverse. According to Zhao et al.^44^, a noteworthy problem is precisely recognizing CKD's presence while ensuring that critical data items from the input dataset are not lost. Furthermore, it has been noted by^37^ that typical Artificial Neural Networks (ANN) are insufficient in terms of their descriptive capabilities for nuanced medical data processing. A deep learning method is employed to address CKD data overtraining.

Nevertheless, given that the Particle Swarm Optimization (PSO) method can gravitate towards local optima owing to its low combination rate. In addition, it has been proven that kernel-based support vector machine (SVM) classifiers tend to result in misclassifications and omissions, notably when dealing with datasets of varied sizes^29,45^. The suggested approach involves the application of BGWO for feature selection and the ELM model for classification. This approach gives a realistic answer by handling concerns with execution time, loss of crucial data surface, and excessive feature dimensions. This result implies substantial progress in accurately predicting CKD and setting new standards in medical data classification.

The proposed work makes the following key contributions:

- This research substantially emphasizes adopting complete data pre-treatment procedures, which involve handling missing values, data transformation, and standardization. Implementing rigorous methods enhances data quality, decreases the consequences of missing values, and ensures that medical data is effectively prepared for analysis.
- This study incorporates feature selection approaches, notably metaheuristic techniques like BGWO, to maximize the model's classification performance. These strategies are critical for refining the input features and ensuring that only the most relevant information is used for classification, thereby enhancing the overall efficiency and accuracy of the model.
- The ELM algorithm assumes a significant role as the principal classification technique. Adopting ELM as a detection tool highlights its applicability and potential for effectively diagnosing CKD. This contribution is pivotal as it applies the prepared and optimized data to identify CKD in patients accurately.
- The strategies for maximizing feature selection and fine-tuning the hidden layer nodes inside ELM are integrated. This approach significantly boosts disease prediction accuracy, offering more accurate and trustworthy CKD diagnoses.
- The proposed approach is subjected to a rigorous examination, encompassing standard metrics such as sensitivity, specificity, accuracy, precision, kappa, and F-score. The study's results demonstrate that the technique achieves a noteworthy degree of accuracy, exceeding the performance of existing classifier models.

## Materials and methods

### Dataset

The model under consideration utilizes the UCI Dataset^46^. This dataset is specifically designed to support studies focused on CKD detection. It comprises 25 attributes, with 14 comprising nominal attributes, 11 comprising numeric attributes, and 1 comprising the class attribute. These attributes include the data of 400 individuals, whereby 250 individuals are categorized as having CKD and 150 individuals are classified as NKD. Table 1 provides a complete summary of the dataset used in this investigation.

**Table 1:** CKD Dataset depiction.

ID (F)	Data Description	Data Type	Unit/Category
F-1	Patient's age	Continuous	Years
F-2	Systolic blood pressure		Mm Hg
F-3	Urine specific gravity	Categorical	Examples: 0.005, 1.010…
F-4	Urine albumin level		Levels:0, 1, 2…
F-5	Urine sugar level		
F-6	Type of red blood cells		Options: Normal, Abnormal
F-7	Type of pus cells		
F-8	Presence of pus cell clumps		Options: Present, not present
F-9	Presence of bacteria		
F-10	Random blood glucose	Continuous	mg/dl
F-11	Blood urea level		
F-12	Serum creatinine level		–
F-13	Blood sodium level		mEq/L
F-14	Blood Potassium level		–
F-15	Hemoglobin level		gms
F-16	Packed cell volume		–
F-17	White cell blood count		Cells/cum
F-18	RBC count		Millions/cubic mm
F-19	Presence of Hypertension	Categorical	Options: Yes, no
F-20	Presence of Diabetes mellitus		
F-21	Coronary artery disease (CAD)		
F-22	Appetite quality		Options: Good, poor
F-23	Pedal edema		Options: Yes, no
F-24	Presence of Anemia		
F-25	Patient classification		CKD, NOT CKD

### Methodology

This study introduces an innovative methodology to address the complexities of forecasting patient ailments. This research aims to advance the precision of disease estimates by applying sophisticated medical data classification methods. Our primary objective is to boost the classification efficacy of the CKD dataset by employing a rigorous feature selection process. In the subsequent parts, we shall explain the comprehensive approach utilized in our study.

Stage. 1 Pre-processing:

In the first step of our inquiry, we apply a dataset regarding CKD provided by the UCI machine learning repository. The admission of noise, incompleteness, and data disputes within real-world medical datasets is generally recognized, principally attributable to these databases’ different sources and sizes. A thorough pre-processing approach is implemented to the CKD dataset to boost the overall data quality. This method involves modifying data, addressing missing data, and normalizing data. This technique not only improves the accuracy of the predictions but also ensures the sanctity of the data set.

Stage. 2 Feature Selection:

A thorough feature selection process must be conducted to achieve the best possible classification accuracy. This study employs the BGWO approach to satisfy the required objective. The BGWO algorithm was pivotal in identifying and selecting the essential data criteria in the available CKD data set. The input for the subsequent classification step comprised carefully chosen features.

**Figure 1:**
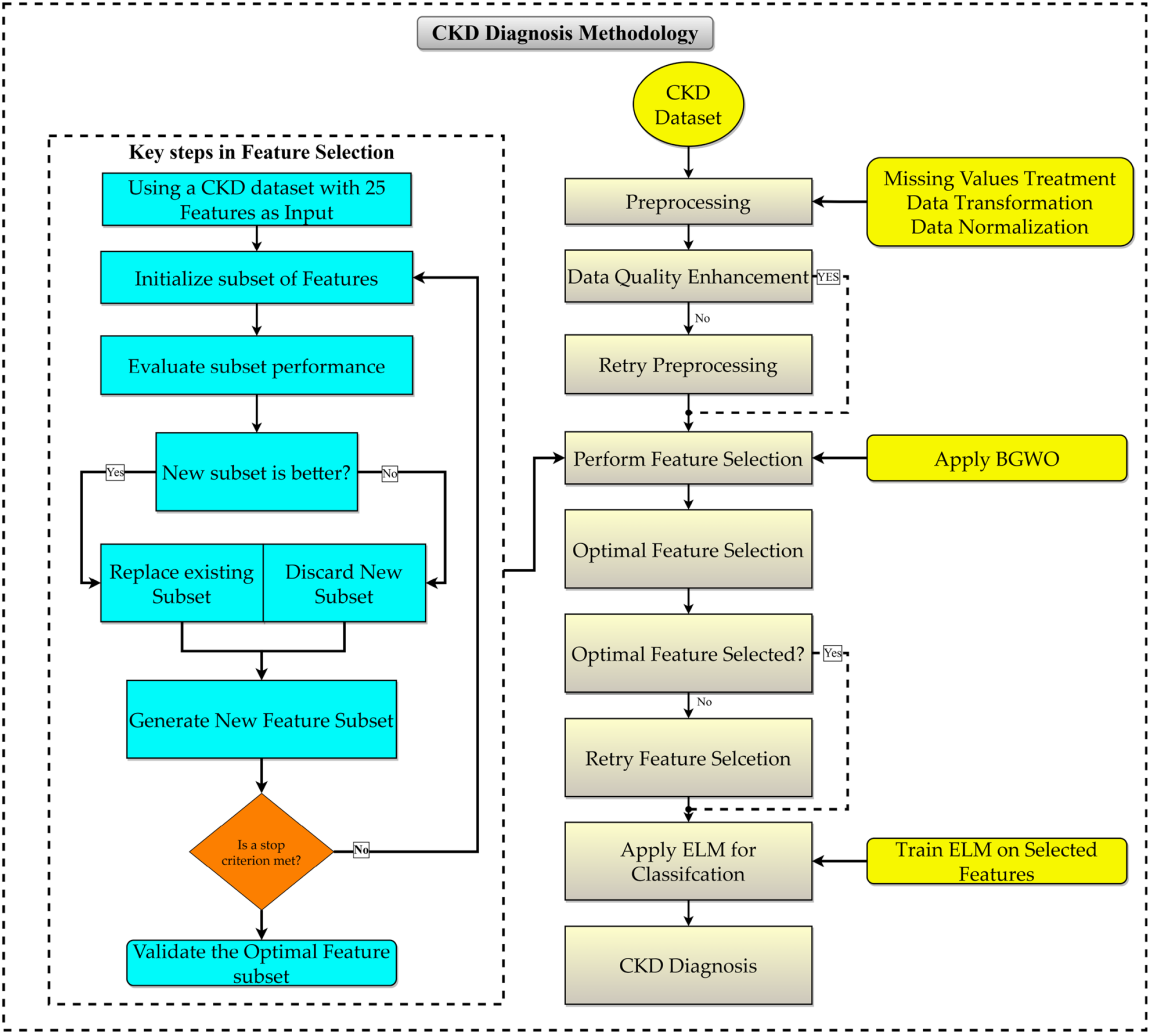
Proposed methodology block diagram.

Stage.3 Data Classification:

ELM was applied in the last stage of the study to classify the existence of CKD. The ELM model uses the criteria provided and runs the learning process to determine whether a person has CKD by using a medical data set. The algorithm has gained universal acceptance due to its outstanding adequacy performance in machine learning and the fact that it can extract complicated patterns from learning machines. This could be attributed to the entities' innate capabilities as they are suitable for classifying medical data, ensuring reliable and robust results. The main objective of the research was to improve the accuracy of CKD diagnosis by a modified method that dealt with the problem of disease classification properly. The objective is accomplished by employing a comprehensive methodology that includes pre-processing methods, optimal feature selection options, and ELM application in classification. The goal was to create an accurate and effective model for detecting CKD from healthcare data sets. At the beginning of the first phase of data preparation, the full process is shown in Fig. 1, and the process ends with classification based on ELM. This method can improve CKD accuracy significantly and change how CKD patient care is offered.

### Data pre-processing

#### Missing value imputation

The dataset offered a major challenge in this inquiry because a considerable portion of the data was missing. As a justifiable solution to this problem, we implemented the Predictive Mean Matching method in the line of multiple imputations. The approach was easily accomplished using the MICE package in R. The programming language's broad functionality allowed us to address the challenge of missing values successfully. The rigorous approach has given us a highly cleaned dataset that was specifically tailored, and a copy was stored in a CSV format. This study used the well-sanitized dataset in the next steps, where we developed different Python models, ensuring the credibility of our predictions and classifications. The details presented below follow the sequential steps outlined in the study^47^.

Sequential steps for PMM are as follows:

**Step 1:** In step one, using a set of regression models, we resolved to estimate, for all subjects with appropriate "*y*" components for which data was missing, the expected means expressed as $"\widehat{yi}"$ . The task also stipulated that computations be conducted to estimate the posterior projected means expressed as ${"yo}^{*"}$ . Herein, "*o*" is a notation considering the context of variables that have data missing.

**Step 2:** In this phase, we selected a set of "*K*" donors that are possibly ideal, with the intended conceptual meaning being that the distance $d\left(0,i\right)=|{yo}^{*}-\widehat{yi}|$ is the minimal. In this context, "donor" denotes variables with no missing values.

**Step 3:** Following the third step of the procedure, a single donor was randomly picked from a list of possible donors. The observed values of the selected donor were subsequently employed to impute the missing values in the recipient's variable "$o$."

#### Data transformation

In the context of our data pre-treatment pipeline, we have handled the conversion of nominal features. These features frequently consist of values that are essentially non-numeric, such as "yes/no," "good/poor," "present/not present," or "normal/abnormal." To facilitate their integration into our analytical methodologies, we transformed them into a binary representation denoted by the digits "1" and "0."

To achieve this change, we utilized the label encoding technique, a function conveniently accessible within the Sklearn package in the Python computer language. This methodology transformed nominal categorical variables into a numerical representation, assuring congruence with the following data analysis and modeling stages.

#### Data normalization

Data normalization is a fundamental approach used in database architecture to organize and structure data to minimize duplication and enhance data quality.

The training and test sets have been standardized to a consistent scale in this stage. Additionally, the data has been adjusted using min–max scaling to achieve a consistent scale. The Equation for min–max normalization, as stated in reference^48^, is denoted as an equation. One throughout the literature.

1 $${X}_{norm}=\frac{{X}_{0}-{X}_{min}}{{X}_{max}-{X}_{min}}$$

The variable ${X}_{norm}$ Signifies the normalized value of variable $X$ following transformation. The symbol ${X}_{0}$ signifies the present value of variable $X$. ${X}_{max}$ Signifies the maximum value inside the dataset. The variable ${X}_{min}$ Signifies the minimum value within the dataset.

#### Dataset splitting

After normalizing the dataset, we partitioned it into two datasets: 80% of the data was used for training, and the remaining 20% was used for testing. The partition was based on the stratified split method. This procedure balanced the cases split for the CKD and non-CKD parameters concerning both datasets. Thus, the model was trained and tested with an actual proportion of classes and had a proportional representation. This ensured the model had an equitable distribution and offered better accuracy and reliability.

### Feature selection

In this module, algorithms use the BGWO method to select a discriminating subset of features according to a certain criterion. Feature selection is a necessary step and forms a method of predominant interest. The traditional feature selection process consists of four main steps: subset formation, subset evaluation, stopping criteria, and previous knowledge or validation of the results. The first step in the subset formation method is to form a candidate feature subset to evaluate, which is generated using the BGWO algorithm based on the processes used by the wolves. Next, each subset formed is evaluated relative to the current subset using a certain criterion. The subset formed is replaced if the new subset formed is better than the previous subset.

This subset formation and evaluation action is repeated until a stopping criterion is met. After this, the subset selected as the most optimum is validated using either previous knowledge or tests on past datasets.

#### Feature subset optimization

Feature selection is a critical aspect of ML, and it significantly impacts dataset quality. The exclusion of unnecessary features adds value by speeding the training time, making model development easier, and improving the understanding of data^49^. Subset optimization is enhanced when the BGWO algorithm is adopted, which helps conceptualize the features of a model as 'Grey Wolves.' Both the interpretability of the model and its performance are enhanced. One of the main reasons why the subset optimization of features is so important is that, in some cases, peaks are reached. This tendency of features reaching maximums increases overfitting, which results in high numbers of features with redundant information. The BGWO algorithm, therefore, has facilitated the increased accuracy of data understanding and the identified features that contribute significantly.

### Grey Wolf optimization algorithm

The approach is a population-based computational optimization technique rooted in evolutionary computing, like the prestamped precession in grey wolves^25^. The acquisition of the social architecture of a grey wolf pack inspired the computational technique. Normally, the pack comprises 5–12 members with comparatively high intelligence. Within the social structure of the group, the grey wolves are classified into four distinct groups, namely alpha ($\alpha$), beta ($\beta$), delta ($\delta$), and omega ($\omega$), based on the prevailing hierarchy. Alpha individuals within a social group are responsible for making predation, rest, and activity choices, whereas beta individuals provide assistance and support. Deltas have a hierarchical relationship with alphas and betas while possessing the ability to exert influence over omegas, who are obligated to comply with the directives of superior wolves.

The model of grey wolf predation has two distinct processes, as stated by^25^. Initially, the wolves encircle the target, as seen by

2 $$\overrightarrow{X}(t+1)={\overrightarrow{X}}_{p}(t)+\overrightarrow{A}\cdot \overrightarrow{D}$$

where the variable $t$ signifies the iterations. The vector $\overrightarrow{X}$ signifies the position of the wolf while ${\overrightarrow{X}}_{p}$ Signifies the position of the target. Additionally, $\overrightarrow{A}$ Refers to the coefficient constant. The vector $\overrightarrow{D}$ is specified by

3 $$\overrightarrow{D}=\left|\overrightarrow{C}\cdot {\overrightarrow{X}}_{p}(t)-\overrightarrow{X}(t)\right|$$

where $\overrightarrow{C}$ Represents the coefficient vector. The vectors $\overrightarrow{A}$ and $\overrightarrow{C}$ are defined by

4 $$\overrightarrow{A}=2a\cdot \overrightarrow{{r}_{1}}-a$$

and

5 $$\overrightarrow{C}=2\cdot \overrightarrow{{r}_{2}},$$

The value of $a$ exhibits a linear drop from a value of two to zero while the number of iterations grows. The vectors $\overrightarrow{{r}_{1}}$ and $\overrightarrow{{r}_{2}}$ Are randomly generated within the range of [0, 1]. Within the framework of the GWO method, the designations of "alphas," "betas," and "deltas" are assigned to the candidate solutions based on their relative performance. Alphas are regarded as the most optimal solution, betas as the second-optimal solution, and deltas as the third-optimal solution. Individuals classified as alphas, betas, and deltas possess a significant amount of knowledge about the location of food resources. Once optimal positions are achieved, it becomes necessary for other search entities, including the omegas, to revise their places as well. To enhance their predatory efforts, wolves must undertake positional updates, especially those occupying the omega position within the pack hierarchy.

6 $$\overrightarrow{X}(t+1)=\frac{\overrightarrow{{X}_{1}}+\overrightarrow{{X}_{2}}+\overrightarrow{{X}_{3}}}{3}$$

where the vectors $\overrightarrow{{x}_{1}}$, $\overrightarrow{{x}_{2}}$ and $\overrightarrow{{x}_{3}}$ Are computed by:

$$\overrightarrow{{x}_{1}}=\left|\overrightarrow{{X}_{\alpha }}-\overrightarrow{{A}_{1}}\cdot \overrightarrow{{D}_{\alpha }}\right|$$

$$\overrightarrow{{x}_{2}}=\left|\overrightarrow{{X}_{\beta }}-\overrightarrow{{A}_{2}}\cdot \overrightarrow{{D}_{\beta }}\right|$$

7 $$\overrightarrow{{x}_{3}}=\left|\overrightarrow{{X}_{\delta }}-\overrightarrow{{A}_{3}}\cdot \overrightarrow{{D}_{\delta }}\right|$$

The first three optimal solutions for every iteration are denoted as $\overrightarrow{{X}_{\alpha }}$, $\overrightarrow{{X}_{\beta }}$, and $\overrightarrow{{X}_{\delta }}$ . The values of $\overrightarrow{{A}_{1}}$, $\overrightarrow{{A}_{2}}$, and $\overrightarrow{{A}_{3}}$ can determined by Eq. (5). The vectors $\overrightarrow{{D}_{\alpha }}$, $\overrightarrow{{D}_{\beta }}$, and $\overrightarrow{{D}_{\delta }}$ This can be derived by:

$$\overrightarrow{{D}_{\alpha }}=\left|\overrightarrow{{C}_{1}}\cdot \overrightarrow{{X}_{\alpha }}-\overrightarrow{X}\right|$$

$$\overrightarrow{{D}_{\beta }}=\left|\overrightarrow{{C}_{2}}\cdot \overrightarrow{{X}_{\beta }}-\overrightarrow{X}\right|$$

8 $$\begin{array}{c}\\ \\ \end{array} \overrightarrow{{D}_{\delta }}=\left|\overrightarrow{{C}_{3}}\cdot \overrightarrow{{X}_{\delta }}-\overrightarrow{X}\right|$$

The vectors $\overrightarrow{{C}_{1}}$, $\overrightarrow{{C}_{2}}$ and $\overrightarrow{{C}_{3}}$ Are designed using Eq. (7) . The process is repeated when the wolves effectively apprehend the prey.

### Binary Grey Wolf optimization

In the context of the GWO algorithm, wolves can dynamically alter their locations to locate and capture prey effectively. However, some tasks, such as feature selection, provide a binary space issue where the solution is constrained to values of either zero or one. This poses a challenge for the conventional GWO algorithm. Therefore, the Binary GWO algorithm has been suggested to conduct feature selection in challenges that include solutions presented in binary form. This study employs two position update methods, namely Position Update Algorithm 1 (PUA1) and Position Update Algorithm 2 (PUA2), as proposed in^25^.

9 $${x}_{d}^{t+1}=\left\{\begin{array}{c}{x}_{1}^{d},\hspace{0.25em}\hspace{0.25em}\hspace{0.25em}\hspace{0.25em} \, {\text{i}}{\text{f}} \, {\text{r}}{\text{a}}{\text{n}}{\text{d}} \, <\frac{1}{3}\\ {x}_{2}^{d},\hspace{0.25em}\hspace{0.25em}\hspace{0.25em}\hspace{0.25em}\frac{1}{3}\le \, {\text{r}}{\text{a}}{\text{n}}{\text{d}} \, <\frac{2}{3}\\ {x}_{3}^{d},\hspace{0.25em}\hspace{0.25em}\hspace{0.25em}\hspace{0.25em} \, {\text{o}}{\text{t}}{\text{h}}{\text{e}}{\text{r}}{\text{w}}{\text{i}}{\text{s}}{\text{e}}\end{array}\right.$$

And

10 $${x}_{d}^{t+1}=\left\{\begin{array}{c}1, \, {\text{i}}{\text{f}} \, {\text{s}}{\text{i}}{\text{g}}{\text{m}}{\text{o}}{\text{i}}{\text{d}} \, \left(\frac{{x}_{1}+{x}_{2}+{x}_{3}}{3}\right)\ge \, {\text{r}}{\text{a}}{\text{n}}{\text{d}} \, \\ 0, \, \, \, \, \, \, \, \, \, \, \, \, \, \, \, \, \, \, \, \, \, \, \, \, \, \, \, \, \, \, \, \, \, \, \, \, \, \, \, \, \, \, \, \, \, \, \, \, \, \, \, \, \, \, \, \, {\text{o}}{\text{t}}{\text{h}}{\text{e}}{\text{r}}{\text{w}}{\text{i}}{\text{s}}{\text{e}} \, \end{array}\right.$$

where $rand$ represents a random number within the range of [0, 1] that conforms to a uniform distribution. The variable ${x}_{d}^{t+1}$ represents the updated position of a $d$-dimensional binary wolf the $tth$ iteration. The sigmoid is formally specified as

11 $$sgimoid(x)=\frac{1}{1+{e}^{-10(x-0.5)}}$$

The variables ${x}_{1}$,${x}_{2},$ and ${x}_{3}$ are binary vectors that symbolize the outcome of wolf movement in the direction of the alpha, beta, and delta grey wolves, respectively. They are designated by

12 $${x}_{1}^{d}=\left\{\begin{array}{c}1,\hspace{0.25em}\hspace{0.25em}\hspace{0.25em}if\hspace{0.25em}\left({x}_{\alpha }^{d}+{bstep}_{\alpha }^{d} \, \right)\ge 1\\ 0, \, \, \, \, \, \, \, \, \, \, \, \, \, \, \, \, \, \, \, \, \, \, \, \, \, \,\,\,\,\,\hspace{0.25em}\hspace{0.25em}\hspace{0.25em}otherwise\end{array}\right.$$

13 $${x}_{2}^{d}=\left\{\begin{array}{c}1,\hspace{0.25em}\hspace{0.25em}\hspace{0.25em}\hspace{0.25em}if\left({x}_{\beta }^{d}+{bstep}_{\beta }^{d}\right)\ge 1\\ 0, \, \, \, \, \, \, \, \, \, \, \, \, \, \, \, \, \, \, \, \, \, \, \, \, \, \, \, \, \, \, \,\,\hspace{0.25em}\hspace{0.25em}\hspace{0.25em}\hspace{0.25em}otherwise\end{array}\right.$$

14 $${x}_{3}^{d}=\left\{\begin{array}{c}1, if \left({x}_{\delta }^{d}+{bstep}_{\delta }^{d}\right)\ge 1\\ 0, \, \, \, \, \, \, \, \, \, \, \, \, \, \, \, \, \, \, \, \,\, \, \, \, \, otherwsise\end{array}\right.$$

The alpha, beta, and delta wolf's positions are denoted as ${x}_{\alpha }^{d}$ , ${x}_{\beta }^{d}$, and ${x}_{\delta }^{d}$ Respectively. Additionally, the values ${\text{bste}}{p}_{\alpha }^{d}$, ${\text{bste}}{p}_{\delta }^{d}$, and ${\text{bste}}{p}_{\beta }^{d}$ are specified by

15 $${bstep}_{\alpha }^{d}=\left\{\begin{array}{c}1, if {cstep}_{\alpha }^{d}\ge rand\\ 0, \, \, \, \, \, \, \, \, \, \, \, \, \, \, \, otherwsise\end{array}\right.$$

16 $${bstep}_{\beta }^{d}=\left\{\begin{array}{c}1, if {cstep}_{\beta }^{d}\ge rand\\ 0, \, \, \, \, \, \, \, \, \, \, \, \, \, \,\,otherwsise\end{array}\right.$$

17 $${bstep}_{\delta }^{d}=\left\{\begin{array}{c}1, if {cstep}_{\delta }^{d}\ge rand\\ 0, \, \, \, \, \, \, \, \, \, \, \, \, \, \, \,otherwsise\end{array}\right.$$

The variables ${\text{cstep }}_{\alpha }^{d}$,${\text{ cstep }}_{\beta }^{d}$, and ${\text{cstep }}_{\delta }^{d}$, are defined as follows.

18 $${cstep}_{\alpha }^{d}=\frac{1}{1+{e}^{-10\left({A}_{1}^{d}{D}_{\alpha }^{d}-0.5\right)}}$$

19 $${cstep}_{\beta }^{d}=\frac{1}{1+{e}^{-10\left({A}_{1}^{d}{D}_{\beta }^{d}-0.5\right)}}$$

20 $${cstep}_{\delta }^{d}=\frac{1}{1+{e}^{-10\left({A}_{1}^{d}{D}_{\delta }^{d}-0.5\right)}}$$

The values of ${A}_{1}^{d}$,${D}_{\alpha }^{d}$ , ${D}_{\beta }^{d}$ and ${D}_{\delta }^{d}$ δ are computed using Eqs. (6), (12), (13), (14).

Similarly, with this BGWO, the data is updated by data (with optimum features) from every place. Algorithm 1 delineates the BGWO pseudocode.

**Figure:**
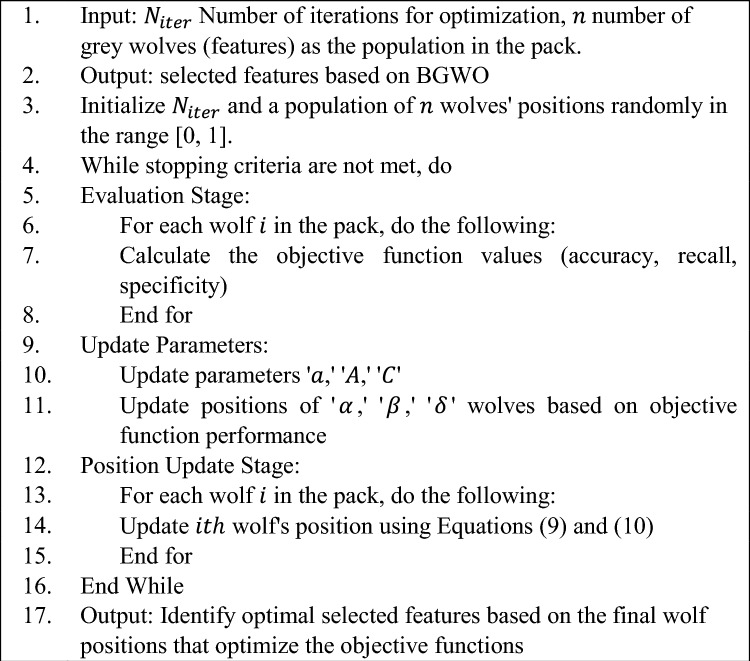
**Algorithm** **1**: The Proposed Pseudocode of (BGWO)

The solution in this research is denoted as a one-dimensional vector, whereby its dimension corresponds to the number of features. In the context of this binary vector, the values 0 and 1 represent the following:

The solution in the present investigation is represented as a vector with one dimension, where the dimension aligns with the number of features. Within the framework of this binary vector, the numerical values 0 and 1 correspond to the following meanings:

0: The feature has not been chosen.

1: The feature has been chosen.

The process of feature selection inherently has a dual-objective aspect. One primary goal is to decrease the number of features, while the other is to improve classification precision. To achieve both goals simultaneously, the fitness function incorporates the following equations, applying the KNN classifier described in^25^ and ^50^.

21 $$fitness=\propto {\rho }_{R}\left(D\right)+\beta \frac{|S|}{|T|}$$

The parameters $\propto$ and $\beta$ are defined as $\alpha =[\text{0,1}]$ and $\beta =(1-\alpha$), respectively, are adopted from^25^.The term ${\rho }_{R}\left(D\right)$ designates the rate of error of the KNN classifier. Furthermore,$|S|$ represents the nominated the features subset, whereas $|T|$ denotes the whole of features included in a data set.

After successfully integrating the optimal feature selection segment, disease detection is conducted using the classifier. The ELM technique utilizes a classification methodology to ascertain the existence or non-existence of CKD by analyzing medical data.

### CKD data classification

The features selected through BGWO are employed in the CKD classification phase. In this phase, we emphasize training an ELM model to classify CKD.

#### Extreme learning machine (ELM)

The ELM^51^ is a highly adaptable feed-forward neural network often employed for many computational tasks, including classification, regression, and clustering. The ELM is capable of having either a single or multiple hidden layers. While a single hidden layer can suffice for simpler problems, providing rapid training and reduced computational demands, it may not perform adequately for more complex datasets, where multiple layers could capture deeper patterns and interactions within the data. The proposed model consists of input notes receptive to the hidden nodes and other notes that form the final output. Similar to other neural networks, rectified linear units activate the hidden nodes. The key feature of our algorithm is that the hidden node parameters are fixed. These parameters include biases and weights. They can either be kept unaltered, or they can be transferred as they are. This differs from the back-propagation algorithm, a common approach used to train neural networks. While effective, back-propagation is limited because weights require continuous updates; the algorithm does not consider the weights' magnitudes and tends to get stuck in local minima. In addition, we included adjustment of weights and biases' magnitude to prevent over-fitting.

**Figure 2:**
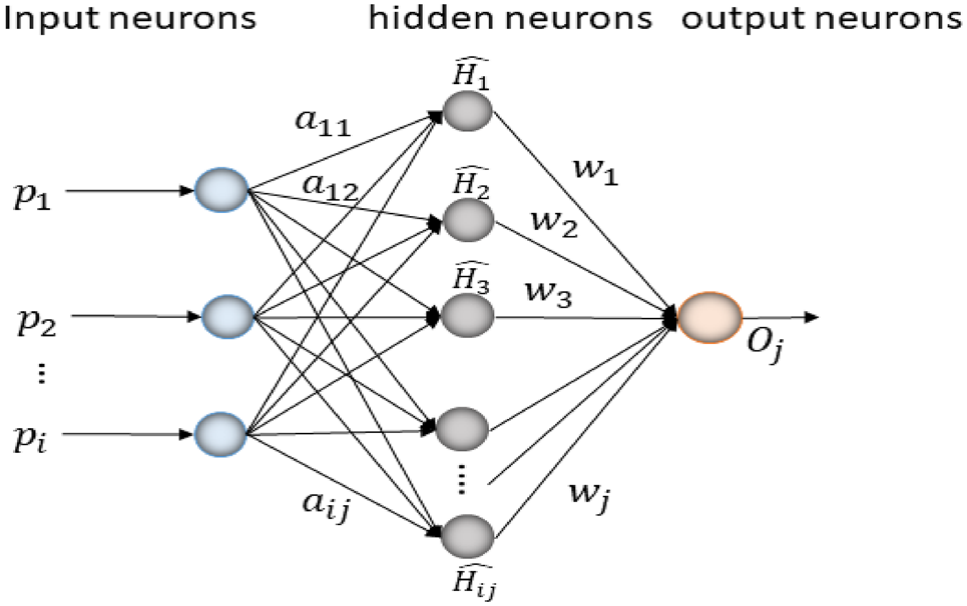
ELM configuration.

The dropout techniques lock the training phase to ensure that the method does not generalize the testing and training around the vectors. However, during testing, all the input node weights are returned & those arriving at the hidden unit nodes are weighted and multiplied. This prevents the number of weights connecting the input and hidden nodes from being changed. The ELM is illustrated in Fig. 2. On the other hand, ELM is better at faster learning when compared to the networks that have been trained on back-propagation. Finally, in using a validation tool, we can watch the learning process and ensure that the complexity of the model allows the model to generalize testing to new data.

Regarding a set of $H$ random samples denoted as ${(pi,t}_{i})$, where ${{p}_{i}=[{p}_{i1},{p}_{i2},\dots ,{p}_{in}] }^{T}\in {Q}^{n}$ and ${{t}_{i}=[{t}_{i1},{t}_{i2},\dots ,{t}_{im}] }^{T}\in {Q}^{m}$ .The basic single-hidden layer feed-forward neural network (SLFN) with $G$ hidden nodes and an activation function $f(.)$ may be mathematically stated as:

22 $$\sum_{i=1}^{G}{w}_{i}f\left({a}_{i}\times {p}_{j}+{c}_{i}\right)={o}_{j}, (j=\text{1,2},\dots .H)$$

The weight vector ${a}_{i}$ linking the $ith$ hidden node with the input nodes, denoted as ${{a}_{i}=[{a}_{i1},{a}_{i2},\dots ,{a}_{in}] }^{T}$ Input nodes are denoted as ${{w}_{i}=[{w}_{i1},{w}_{i2},\dots ,{w}_{in}] }^{T}$.In this context, this weight vector links the $ith$ hidden node to the output node. The variable ${c}_{i}$ Signifies the threshold value related to the $ith$ hidden node. Additionally, the variable ${{o}_{j}=[{o}_{j1},{o}_{j2},\dots ,{o}_{jn}] }^{T}$ Signifies a vector of outputs for the $jth$ node, which is created by the SFFN.

Within the SLFN domain that uses $G$ hidden nodes and an activation function $f(.)$, these networks can accurately estimate a collection of $H$ illustrations without error. The condition ${\sum }_{j=1}^{G}\parallel {o}_{j}-{t}_{j}\parallel =0$ represents an accurate estimate, indicating that the total of the discrepancies between the output values ${o}_{j}$ of the network and their respective goal values ${t}_{j}$ It is equal to zero. This noteworthy accomplishment is made possible by the presence of appropriate weight vectors.${w}_{i}$, input vectors ${a}_{i}$, and hidden node thresholds ${c}_{i}$, which guarantees the fulfillment of this zero-error criterion.

23 $$\sum_{j=1}^{G}{w}_{i}f\left({a}_{i}\times {y}_{i}+{c}_{i}\right)={t}_{j} (j=\text{1,2},3,\dots H)$$

The Equation mentioned above may be concisely stated as below:

24 $$Mw=T$$

where

25 $$M\left({a}_{1},\dots ,{a}_{G},{c}_{1},\dots ,{c}_{G},{y}_{1},\dots ,{y}_{G}\right)={\left[\begin{array}{ccc}f\left({a}_{1}\times {y}_{1}+{c}_{1}\right)& \cdots & f\left({a}_{G}\times {y}_{1}+{c}_{G}\right)\\ \vdots & \cdots & \vdots \\ f\left({a}_{1}\times {y}_{H}+{c}_{1}\right)& \cdots & f\left({a}_{G}\times {y}_{H}+{c}_{G}\right)\end{array}\right]}_{H\times G}$$

26 $$w={\left[\begin{array}{c}{w}_{1}^{T}\\ \cdot \\ \cdot \\ \cdot \\ {w}_{N}^{T}\end{array}\right]}_{G\times n}$$

27 $$T={\left[\begin{array}{c}{t}_{1}^{T}\\ \cdot \\ \cdot \\ \dot{{t}_{N}^{T}}\end{array}\right]}_{G\times n}$$

The term "$M"$ represents the output matrix derived from the hidden layer. In matrix $M$, each column, expressed as the $kth$ column, corresponds to the output produced by the $kth$ hidden node concerning the inputs ${y}_{1}$,${y}_{2}$ and so forth up to ${y}_{H}$. The resolution of the linear system may be mathematically epitomized as:

28 $$w={M}^{-1}T$$

In the given context, the symbol ${M}^{-1}$ denotes the Moore–Penrose generalized inverse of the matrix $M$.

The ELM's output function is defined as below:

29 $$g(y)=p(y)w=p(y){M}^{-1}T$$

In the context of ELM training, three vital parameters are of significance. These parameters include the training set, which is signified as $K=\left[\left({y}_{j},{t}_{j}\right)\right| {y}_{j}\in {Q}^{n}, {t}_{j}\in {Q}^{m}, j=\text{1,2},\dots ,H]$, the output function of hidden nodes, denoted as $f({a}_{i},{c}_{i},{y}_{i})$, and the number of hidden nodes, referred to as $G$. The ELM training procedure may commence once all parameters have been properly set.

The Extreme Learning Machine starts its training process by generating random values for the *G* pairs of hidden node parameters ${a}_{i},{c}_{i}$.. The output matrix *M* is then created using Eq. (24). Since the model constitutes input data along with these randomly generated parameters, the ELM can then evaluate the output weight vector *w* with the help of Eq. (28). Once the training process is completed, the model can be applied to predict the results for the test data tuples using Eq. (29). In this way, the ELM training process can be defined as follows:

The training set A is provided by $A=\left\{\left({a}_{i},{d}_{i}\right)|{a}_{i}\in {X}_{n}, {d}_{i}\in {X}_{m}, i=\text{1,2},\dots N\right\}$ with activation function $f(x)$ and the number of hidden neurons $N$:

Initially, random values are assigned to the input weights ${w}_{i}$ and biases ${b}_{i}$.

Then, a computation is held to determine the resulting matrix *M* of the hidden layer.

The output weight vector *w* can be computed as follows: $w=M\times T$

This structured training process allows ELM to effectively analyze and classify the collected data, thereby allowing accurate predictions of the findings for any new samples.

## Results and discussion

### Experimental setup

The presence of chronic kidney disease (CKD) in the dataset was determined using BGWO feature selection, and the ELM classifier was implemented in MATLAB/Simulink on a workstation equipped with an Intel Core i7, 2.60 GHz CPU and 8 GB RAM. The performance metrics, namely accuracy, recall, specificity, kappa, and F-score, were evaluated, and the actual results were compared with the predicted outcomes.

The following information outlines the fundamental parameter configurations. The method for BGWO was implemented with a population size of 15 wolves, aiming to attain a harmonious equilibrium amid exploration and exploitation strategies. A maximum of 100 iterations was set. In the ELM scenario, we conducted experiments to evaluate the impact of varying the number of hidden nodes ranging from 50 to 250. Additionally, we applied the sigmoid activation function to enhance the classification capabilities of the model.

### Evaluation criteria

The key purpose of this study is to ascertain the classification of an input sample as either a positive sample class or a negative sample class. Four potential estimation outcomes exist, which may be classified according to the nomenclature outlined as follows:

- True Positive (TP) – the model accurately predicts the positive class.
- True Negative (TN) – the model accurately predicts the negative class.
- False Positive (FP) – the model accurately predicts the positive class.
- False Negative (FN) – the model inaccurately predicts the negative class.

Table 2 provides the formulas for various estimation measures used in our analysis.

**Table 2:** Metrics for Assessing Classification Model Performance.

Formula	Expected values
$Accuracy=\frac{{t}_{p}+{t}_{n}}{Total data instances}$	High
$Recall=\frac{{t}_{p}}{{t}_{p}+{f}_{n}}$	High
$Specificity=\frac{{t}_{n}}{{t}_{n}+{f}_{p}}$	High
$Kappa=\frac{{p}_{0}-{p}_{a}}{1-{p}_{a}}$	A value of 1 designates complete agreement, while a value below 1 implies a lesser degree of agreement
$F-Score=\frac{2\times Recall\times Precision}{(Recall+Precision)}$	The best value is 1, while the worst value is 0

Classification Accuracy: This statistic quantifies the ratio of accurately categorized data tuples to the overall number of classifications.

Recall (Sensitivity): The recall metric estimates the ratio of correctly anticipated positive instances to the overall number of positive cases.

Specificity: Specificity assesses the proportion of true negative outcomes appropriately classified by the classifier concerning the overall number of predicted negative outcomes. It is particularly useful in scenarios where correctly identifying negative cases is vital.

Out of the 25 features in the CKD dataset, this study utilized Binary BGWO to select the optimal subset of 15 features. The chosen features and their descriptions are detailed in Table 3.

**Table 3:** Acquired optimal features by BGWO algorithm.

Attributes label	Attributes description
Age	Patient's age
Blood pressure	Indication of heart rate
Specific gravity	The ratio of urine density to water density
Red blood cells	Amount of red blood cells in urine
Bacteria	Identification of kidney infection, if present, and bacterial levels
Blood glucose random	Measurement of random blood glucose (sugar) levels
Blood urea	Amount of urea nitrogen in the blood
Serum creatinine	Presence of creatinine in blood cells
Sodium	The volume of sodium levels in the blood
Potassium	Quantity of potassium levels in the blood
Hemoglobin	Protein in red blood cells, with specific details
White blood cell count	Calculation of the number of white blood cells in the blood
Hypertension	Indication of high blood pressure
Coronary artery disease	Detects the presence of heart disease that may affect kidney function
Appetite	Measures the detection of appetite loss

### Performance Evaluation of Feature Selection Algorithms

Within this particular section, a thorough examination was undertaken to assess the effectiveness of numerous feature selection methods. The accuracy of these algorithms was assessed across different feature set sizes ranging from 10 to 60, using the CKD database as our testbed.

**Figure 3:**
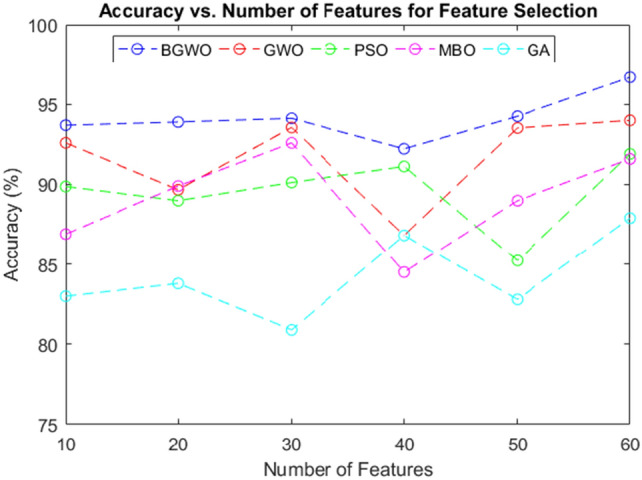
Accuracy analysis of feature selection algorithms.

Figure 3 In this figure, we showcase the accuracy results of various algorithms, namely the proposed BGWO, GWO, Particle swarm optimization (PSO), Monarch Butterfly Optimization (MBO), and Genetic Algorithm (GA), across varying numbers of features in the CKD dataset. The accuracy assessments were conducted based on the selected features. Table 4 provides a detailed overview of the simulation outcomes for several feature selection algorithms.

**Table 4:** Performance analysis comparing the proposed feature selection algorithms with existing methods for predicting CKD.

Algorithm	Accuracy	Recall	Specificity
BGWO	98.95	98.45	93.50
GWO	95.44	94.35	93.32
PSO	93.62	91.45	90.22
MBO	90.32	87.12	91.55
GA	88.35	85..42	89.32

This study's findings reveal that the BGWO model consistently performed better than other feature selection methods, achieving notably high accuracy on the CKD dataset.

### Performance analysis of classification techniques

In this phase of our study, we executed several classification techniques, including AdaBoost^52^, Naïve Bayesian (NB)^53^, Perceptron^54^, and k-Nearest Neighbors (KNN)^55^. Subsequently, we evaluated the efficacy of the ELM model.

#### Enhancing ELM performance through variations in hidden nodes

**Figure 4:**
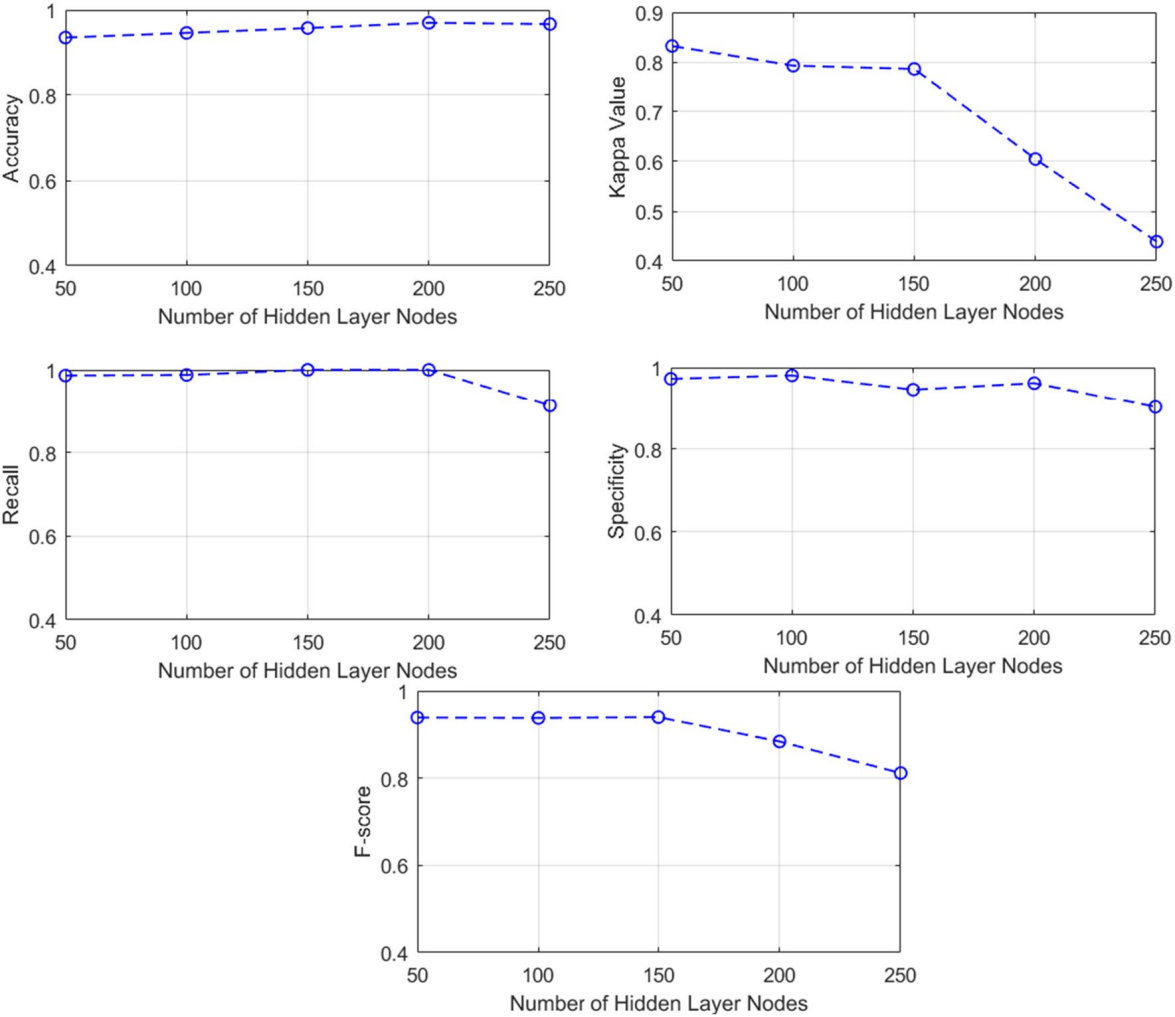
Graphical representation of the ELM efficacy.

To enhance the accuracy of the ELM, the authors conducted experiments involving the adjustment of the number of nodes in the ELM model's hidden layer. The range explored for the hidden layer node count extended from a minimum of 50 to a maximum of 250. The experimentation revealed that modifying the number of hidden layer nodes led to a notable efficiency improvement in the ELM model. For a summarized presentation of the results, please refer to Table 5, and for a visual depiction, consult Fig. 4.

**Table 5:** Comparative Analysis of ELM Performance across Varied Hidden Layer Nodes.

Number of Hidden Layer Nodes	50	100	150	200	250
Accuracy	0.9351	0.9461	0.9575	0.9698	0.9668
Kappa	0.8322	0.7927	0.7860	0.6050	0.4389
Specificity	0.8960	0.8939	0.8861	0.7921	0.7298
Recall	0.9865	0.9878	1.0	1.0	0.9128
F-Score	0.9389	0.9380	0.9398	0.8840	0.8115

The data shown in Table 5 demonstrates that the ELM attains the best level of accuracy when using 200 hidden nodes. Configurations utilizing 250, 150, 100, and 50 nodes also yield relatively high levels of accuracy, although somewhat lower than the configuration mentioned above. While the rise in the number of nodes from 50 to 200 results in a little fall in the Kappa value, it is noteworthy to mention a major improvement in the recall and F-score values. The ELM model demonstrates the highest accuracy (0.9698) when using 200 hidden layer nodes. This is followed by configurations with 250 (0.9668), 150 (0.9575), 100 (0.9461), and 50 (0.9351) hidden layer nodes, respectively. The distribution of input processing over several neurons via an increase of hidden layer nodes reduces the computational burden per neuron and streamlines operations. Nevertheless, when the number of nodes is above a certain threshold, it gives rise to intricacies in the input handling process, leading to a decline in the model's overall performance^56^.

#### Performance comparison of ELM with different classification models

**Figure 5:**
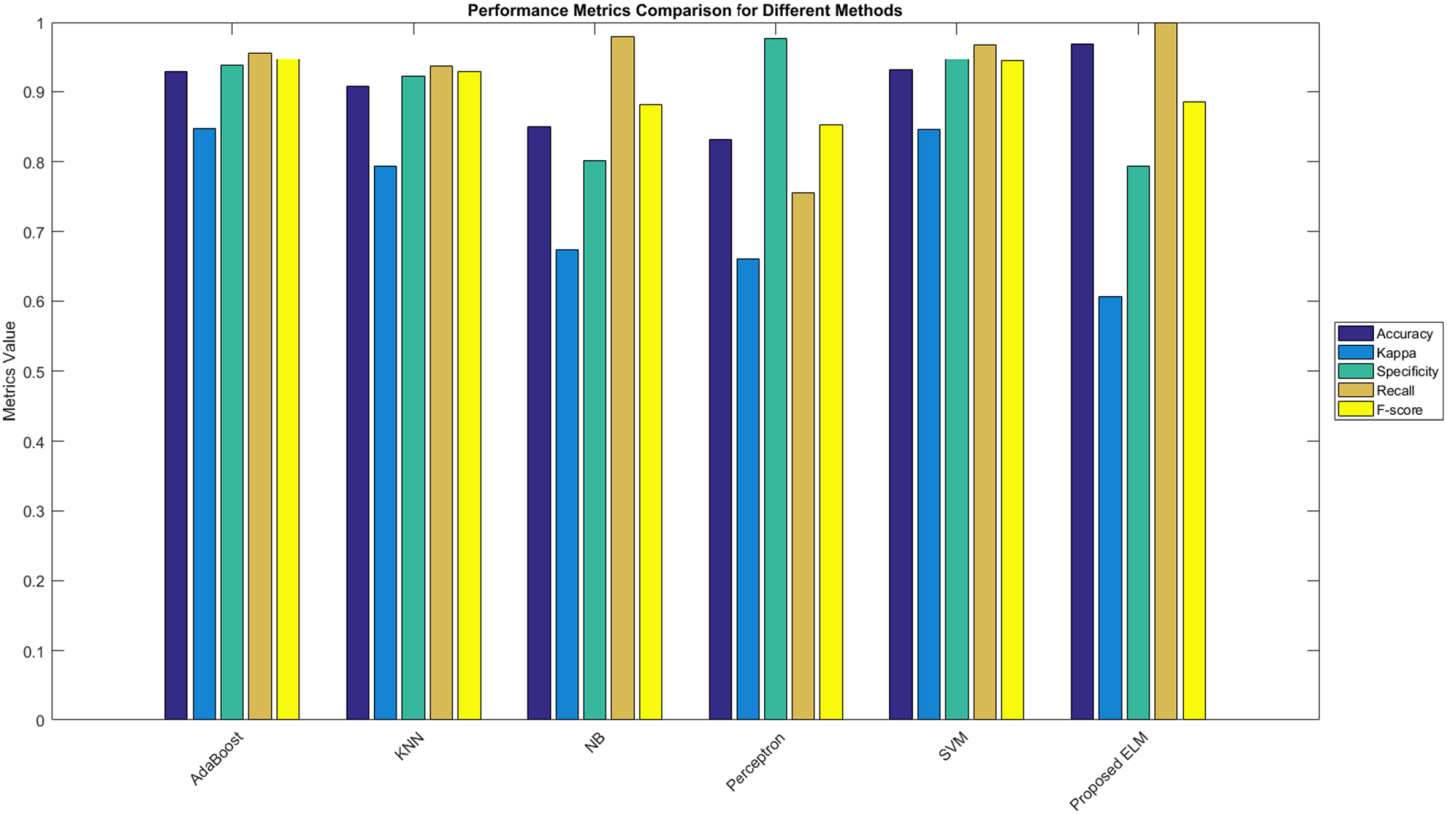
Comparative analysis of CKD medical data set classification algorithms.

This section compares the performance of many classifiers compared to the ELM classifier, which has been constructed with 200 nodes. It is important to acknowledge that the dataset has been partitioned into two distinct subsets, whereby 80% of the tuples are designated for training. In contrast, the remaining 20% are put aside for testing. Multiple evaluation criteria, such as accuracy, Kappa statistics, specificity, recall, and F-score, were employed to facilitate comparison. The results are briefly presented in Table 6, accompanied by a graphical depiction in Fig. 5.

**Table 6:** Classification results of the proposed and existing algorithms.

	AdaBoost	KNN	NB	Perceptron	SVM	Proposed ELM
Accuracy	0.9288	0.9075	0.8498	0.8314	0.9312	0.9698
Kappa	0.8470	0.07933	0.6748	0.6610	0.8457	0.6066
Specificity	0.9380	0.9222	0.8010	0.9775	0.9474	0.7932
Recall	0.9555	0.9365	0.9798	0.7550	0.9674	1.000
F-score	0.9466	0.9280	0.8818	0.8523	0.9444	0.8854

According to the data shown in Table 6, the ELM model has the greatest level of accuracy, measuring at 0.9698. Conversely, the Perceptron model exhibits the lowest accuracy, scoring 0.8314. After thoroughly analyzing many models, it became apparent that the suggested ELM exhibited superior performance compared to all traditional classification approaches.

**Figure 6:**
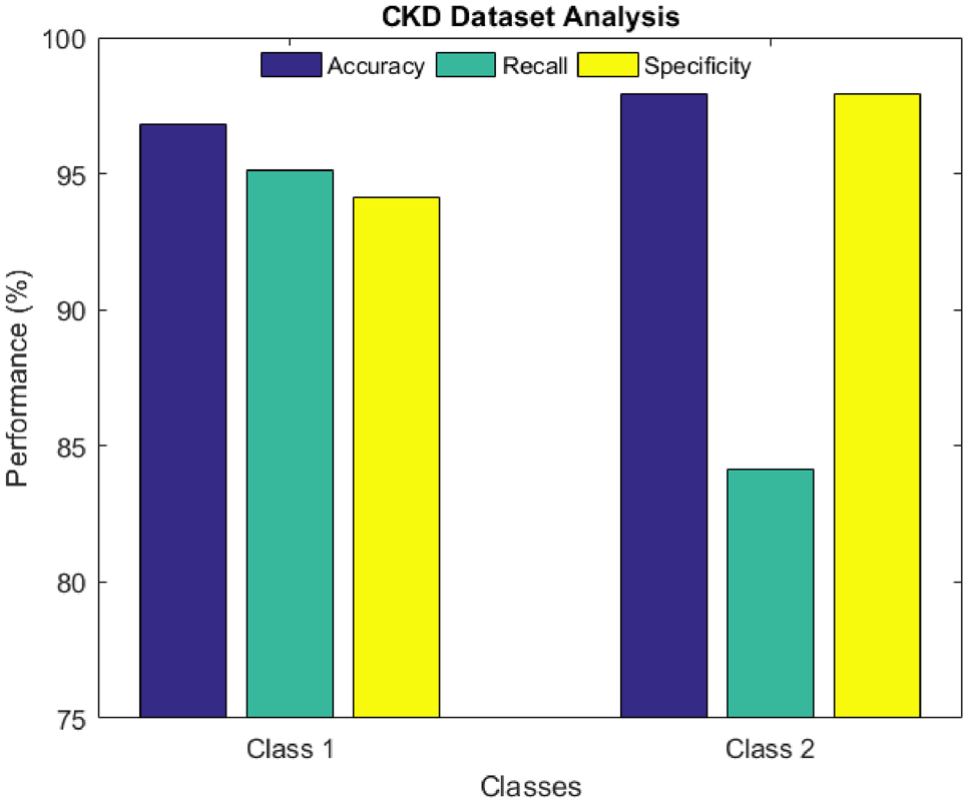
CKD dataset analysis.

The analysis of the CKD dataset is shown in Fig. 6. This dataset consists of two unique classes, class 1 and class 2, which indicate the existence or non-existence of CKD. The graph displays the classes on the x-axis and the performance measures, which include the accuracy, recall, and specificity on the y-axis. The recommended technology was incredibly useful as it yielded impressive results. More specifically, the statistics acquired from the assessment of class 1 showed that the accuracy reached 96.8%, the sensitivity 95.10%, and the specificity 94.12%. Additionally, class 2 was assessed, and results similar to the ones received when examining class 1 were gained, with the accuracy at the 97.90% mark, the sensitivity at 84.15%, and the specificity at 97.90%.

The Receiver Operating Characteristic curve is a graphical standpoint used in the analysis of classifiers' diagnostic skills as well as feature selection strategies. The ROC curve represents the trade-off between sensitivity, defined as the True Positive Rate, and 1-Specificity, the False Positive Rate. Meanwhile, the Area Under the Curve represents a critical parameter of the efficacy of a classification model—the closer the AUC is to 1, the more accurate the model is. ROC curves were plotted in a particular study utilizing the Chronic Kidney Disease dataset. The study was aimed to test the effectiveness of different feature selection strategies. Specifically, these strategies were Genetic Algorithm, Monarch Butterfly Optimization, Particle Swarm Optimization, Grey Wolf Optimizer, and Binary Grey Wolf Optimizer, along with two different classifiers: Extreme Learning Machine and Support Vector Machine. The AUC values provided for each combination give substantial insights into the efficiency of each feature selection approach in specific relationships with classifiers.

- With the ELM classifier, the AUC values were:
- GA: 0.965 ± 0.008
- MBO: 0.966 ± 0.008
- PSO: 0.982 ± 0.006
- The AUC values for GWO and BGWO were not specified but are implied to be significant.
- With the SVM classifier, the AUC values were:
- GA: 0.964 ± 0.008
- The AUC values for MBO, PSO, GWO, and BGWO were 0.966 ± 0.007, with BGWO achieving the highest score.

**Figure 7:**
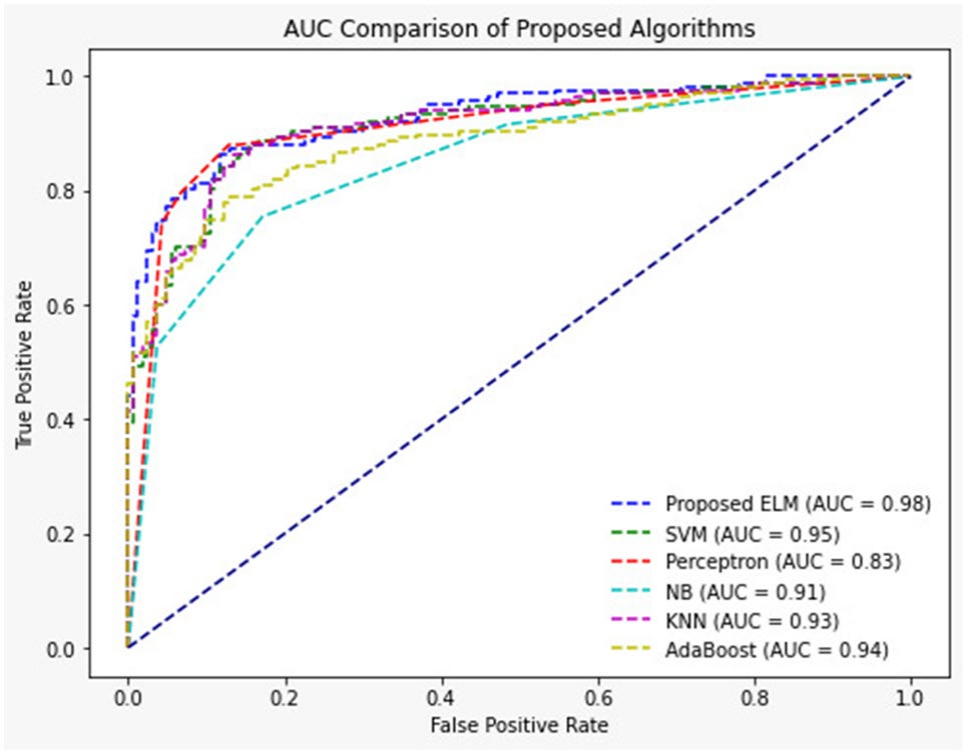
ROC.

From the conducted research, it was proven that such an approach as BGWO is efficient. Thus, according to the ROC curve given in Fig. 7, when BGWO was implemented with the ELM classifier, the AUC value was equal to 0.982 ± 0.006, which means that this approach can optimize categorization better. This study shows that BGWO can be regarded as a useful feature selection strategy to improve the performance of classifiers and enhance the diagnosis accuracy.

### Execution time analysis

**Figure 8:**
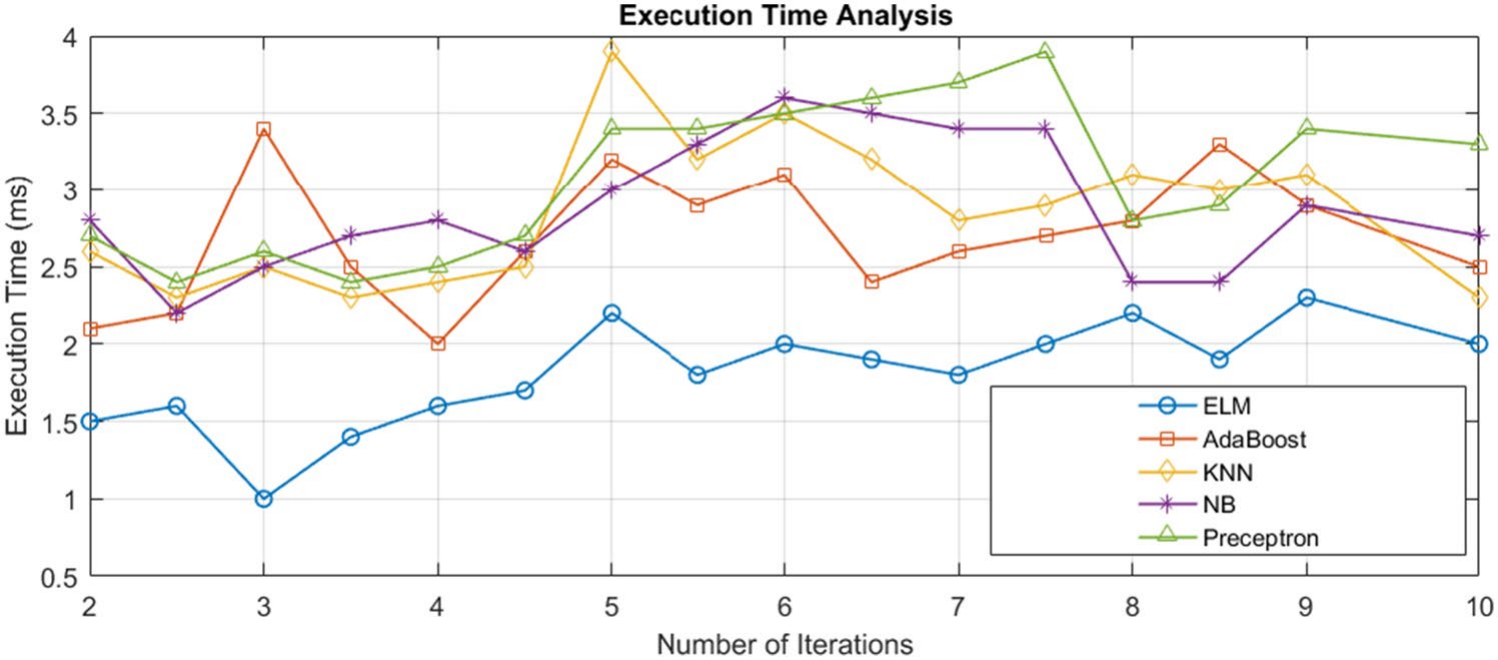
Execution time analysis.

Given the forecasting of the actualization of CKD-influenced persons, we looked into the span of various tasks, as portrayed in Fig. 8. In our work, we first analyzed the computational ability of different machine learning algorithms such as AdaBoost, KNN, NB, Perceptron, SVM, and our proposed method, ELM. The simulation analysis started by applying pre-processing tasks on the input dataset. Following this, the BGWO method was employed to quantify each data set's features. Applying this feature selection technique was essential to facilitate our ability to reach the highest level of accuracy during our simulations while reducing the processing time required.

On the other hand, the BGWO process was adopted to rank the subset of features and select the best-rated ones. The features given above were then fed into the classifier for individual consideration. At the time of this analysis, we regularly monitored the application of the classification rate, aspiring for an elevated level of accuracy, specificity, and recall levels, which can be noticed in Fig. 5.

### Computational complexity analysis

This section considers computational aspects involved in an advised method for CKD categorization. The solution employs the BGWO algorithm for feature selection and the ELM method for classification. The pre-processing step is performed effectively, demonstrating a linear time-based complexity of $O\left(n\right),$ where *'n'* is a picture's original dimensionality. The BGWO algorithm utilized for performance improvement has a time complexity $O(k\times n\times m)$ with '*k'* indicating the number of iterations, '*n'* is the picture's original dimensionality, and 'm' used to describe the feature space size. The ELM model for CKD classification shows a linear complexity of time utilized for the original picture pre-processing represented by $O(p\times \left(n+m\right))$. Here, '*p'* refers to the hidden neurons number, '*n'* serves as the dimensionality of the pre-processed picture, and '*m'* is the feature selection model size. As a result, the BGWO-ELM calculation model employed to address CKD diagnostic problems accepts a solution complexity of $(O\left(k\times n\times m+p \times \left(n+m\right)\right))$. The latter demonstrates the current model is more computationally efficient and precise than the former methods.

## Discussion

Evaluating feature selection algorithms and classification models in diagnosing Chronic Kidney Disease results in a highly rich and diverse locus of research into the viable possibilities offered by modern machine learning models in medical diagnostics. For this purpose, the work centers around a comparative analysis of feature selection algorithms. To determine the mentioned algorithms' robustness of their feature selection capabilities, data are considered at different sizes of feature sets—from 10 to 60—using the CKD database. Notably, the mentioned algorithm appears to be an effective strategy, as it consistently offers higher accuracy, recall, and specificity levels. This conclusion is noteworthy because it accentuates the BGWO approach's valuable, previously undiscovered benefits for increasing the overall accuracy of diagnostic models in cumbersome datasets such as the CKD database. The research also considered multiple classification approaches, including AdaBoost, Naïve Bayesian, Perceptron, k-Nearest Neighbor, SVM, and ELM. The critical aspect of this research includes adjusting the ELM model by varying the number of hidden nodes. The results identified this number as being at the maximum level of 200, as it showed the highest performance levels achievable by the model. The ELM classifier also showcased the highest level of accuracy in comparison with the remaining classifiers, which is especially true for the number of hidden nodes highlighted as displaying optimal accuracy. These data highlight that the ELM model is a highly effective classifier for managing the intricacies present in the CKD datasets when adjusted to the specified parameters.

The research data can also be deepened by applying the Receiver Operating Characteristic curve and the Area Under the Curve parameter, which is critical in evaluating the applied models' diagnostic accuracy. The data is relevant in the case of the ELM classifier, and the results show that the AUC values are high, which is strongly evident for the BGWO algorithms. The high AUC values strongly denote the high diagnostic abilities of the considered models, presenting them as highly viable options in clinical situations where high levels of accuracy are often of primary importance. Altogether, the data showcases how, due to the efforts of this research, a variety of intricate aspects of feature selection and classification models in medical diagnostics are unveiled. It highlights how the research accentuates the value offered by certain approaches, namely BGWO and ELM, to increase the accuracy and efficiency of CKD diagnosis. Finally, it is relevant to the clinical implementability of the research results, as they offer actual benefits for clinical practitioners, who may improve kidney disease patients' health outcomes through early and high-precision diagnosis.

## Conclusion

This study analyzes medical data and categorizes methodologies to identify the early stages of an illness. In particular, it focuses on chronic kidney disease and, more specifically, difficulty in feature selection from the CKD dataset to determine the most critical subset. The methodology included a pre-processing step that addressed the issue of missing values in the dataset, namely, data transformation and data standardization. Using the Binary Gray Wolf Optimization method determines the optimality of the process. After that, the dataset is divided into two categories: the presence of CKD and its absence, denoted by the specified corresponding characteristics. At the classification stage, it is proposed that the Extreme Learning Machine be used. To achieve optimal performance and efficiency, the process of ELM classification was optimized by varying the number of hidden nodes to find a balance between the number of nodes and the accuracy of data processing. As a result, the optimization of performance characteristics, such as sensitivity, specificity, and accuracy, can be finalized by ELM, achieving the highest values of these characteristics. The study's data suggest that the classification accuracy of the ELM method is at a maximal value of 98.90%, which is significantly higher than that of other methods.

Future studies will include improvements to the Binary Gray Wolf Optimization algorithm structure, such as defining a fuzzy or improved BGWO algorithm. It is also possible to apply the proposed methodology to a larger number of datasets from various sources and contact hospital management to obtain a more diverse dataset that would be more robust and likely to include a range of clinically relevant samples. This will make the proposed framework more universal and help solve a wider range of issues than the CKD diagnosis.

## Data Availability

The datasets analyzed during the current study are available in the UCI machine learning repository, LINK: https://archive.ics.uci.edu/dataset/336/chronic+kidney+disease.
